# Comparison of the effects of amantadine and ondansetron in treatment of fatigue in patients with multiple sclerosis

**DOI:** 10.1186/s40169-019-0239-4

**Published:** 2019-07-01

**Authors:** Mojtaba Khazaei, Ashkan Karevan, Mohammad Taheri, Soudeh Ghafouri-Fard

**Affiliations:** 1 Neurophysiology Research Center, Hamadan University of Medical Sciences, Hamadan, Iran; 2 Urogenital Stem Cell Research Center, Shahid Beheshti University of Medical Sciences, Tehran, Iran; 3 Department of Medical Genetics, Shahid Beheshti University of Medical Sciences, Tehran, Iran

**Keywords:** Multiple sclerosis, Fatigue amantadine, Ondansetron, Clinical trial

## Abstract

**Background:**

Multiple sclerosis (MS) is a common neurological disorder with a variety of manifestations including fatigue. Fatigue may interfere with daily work and activities. Different pharmacological and non-pharmacological methods have been used for treatment of this symptom in MS patients. In this study, the effect of ondansetron and amantadine in the treatment of fatigue was compared.

**Methods:**

In this randomized clinical trial, 53 MS patients with fatigability were enrolled (mean age ± standard deviation: 54.00 ± 7.88, Female/male ratio: 45/8). Patients were referred to Imam Clinic and Sina Hospital, Hamadan, Iran. Patients were assessed using the Fatigue Severity Scale (FSS) questionnaire. Patients were randomly assigned to either the amantadine or ondansetron treatment groups and received treatments in a crossover manner. The severity of fatigue was measured using FSS questionnaire in four stages (beginning and end of each regimen). Data were analyzed using SPSS software version 16.

**Results:**

The mean and standard deviation of patients’ fatigue scores before treatment were 43.07 ± 10.36 and 43.22 ± 9.67 in the amantadine and ondansetron group, respectively. These scores were 37.36 ± 7.87 and 40.00 ± 8.94 after treatment in the amantadine and ondansetron group, respectively. Both drugs significantly decreased the fatigue severity of patients (P < 0.001). There was no statistically significant difference between two regimens in terms of the mean score of fatigue before and after treatment and the frequency of complications. However, when ranking the severity of fatigue (mild, moderate, severe), fatigue reduction after intervention in the amantadine group was significantly higher than ondansetron (P = 0.026).

**Conclusion:**

Both amantadine and ondansetron reduce fatigue in MS patients, but the efficacy of amantadine in reducing the MS-associated fatigue is greater than that of ondansetron.

## Introduction

Multiple sclerosis (MS) is a demyelinating disorder of central nervous system which affects more than 2.5 million persons in the world [1]. The disease has a relapsing–remitting nature. Common therapeutic options for MS can reduce the incidence of relapses and formation of plaques. Yet, these therapeutic methods fail to ameliorate established brain lesions or chronic symptoms including fatigue, which is a frequent observed symptom in all MS subtypes [1]. Fatigue compromises all four domains of quality of life namely physical health, psychological, environmental, and social relationships in MS patients [2]. The decreased quality of life due to fatigue is independent of MS-associated depression or debility [3]. In several patients, fatigue is stated as the single most incapacitating symptom, even more than pain and physical debility [4]. Fatigue is scored based on different scales including fatigue severity sale (FSS) [5]. Numerous pharmacological and non-pharmacological strategies have been developed for amelioration of this MS-associated symptom [6]. Amantadine as an antiviral drug is one of the suggested interventions for management of fatigue. Although not clearly defined, amantadine might affect MS-associated fatigue through its antiviral function, immune-mediated mechanism, or an amphetamine-like activity [7]. A double-blind crossover trial in MS patients has shown decreases in FSS following treatment with both amantadine and aspirin with no significant inter-group difference [8]. Another blinded, placebo-controlled trial reported significant differences in the Modified Fatigue Impact Scale (MFIS) scores between amantadine-treated patients and placebo-received ones [9]. Ondansetron, a 5-HT3 receptor antagonist has been tested for treatment of chronic nausea and vertigo in MS patients [10] and for treatment of chronic hepatitis C-associated and biliary cirrhosis-associated fatigue [11, 12]. In the current randomized clinical trial, we compared the effects of amantadine and ondansetron on MS-associated fatigue. The objectives of the current study were evaluation of the effects of these drugs in Iranian MS patients and comparison between these drugs by using the Persian version of FSS. These drugs were chosen based on the results of previous studies indicating the effectiveness of amantadine and ondansetron in ameliorating fatigue in MS and cirrhosis, respectively. Moreover, as ondansetron has been useful for management of chronic nausea and vertigo in MS patients, if its effects on fatigue were comparable with commonly used anti-fatigue drugs in MS such as amantadine, it could be used as a treatment for MS patients who suffer from these three symptoms. The dose and the time of administration of amantadine (100 mg twice a day for 4 weeks) were selected based on the results of previous clinical trials in MS patients [7]. For ondansetron, the dose and time of administration were similar to the results a clinical trial in chronic hepatitis C patients [13].

## Methods

### Patients

The current clinical trial was conducted on patients referred to Imam Clinic and Sina Hospital, Hamadan, Iran during November 2018–March 2019. The severity of fatigue was scored based on the Persian version of FSS. The validity and reliability of Persian version of this scoring system had been approved previously [14]. Inclusion criteria for MS patients were age between 18 and 65 years, fatigue complaint, the ability to walk without assistance of at least 100 m, FSS score ≥ 28. Exclusion criteria were systemic diseases such as cardiovascular diseases, infection, thyroid disease, or vasculitis, taking other drugs that affect fatigue symptom (beta blockers, antidepressants, sedatives, modafinil, Ritalin or pemoline) in the prior 6 months period, risk factors for long QT, electrolyte imbalance, close-angle glaucoma or pregnancy.

Consent form was filled out by all the study participants and the study protocol was approved by the local Ethical committee of Hamadan University of Medical Sciences (IR.UMSHA.REC.1397.500). The clinical trial registry number was IRCT20120215009014N250.

### Study design

This was a randomized crossover clinical trial. Non-probability sampling method (convenience sampling) was used. Sixty-one MS patients were randomly assigned to two groups receiving ondansetron (4 mg twice a day) or amantadine (100 mg twice a day) for 4 weeks. Fatigue was assessed using FSS. After a two-week discontinuing of drugs, patients received the other drug for another 4 week period. Any side effect of drugs was assessed to conduct appropriate interventions if needed.

### Measured outcomes

Fatigue score before and 4 weeks after treatment.

### Statistical methods

Data were analyzed using SPSS v. 16 (IBM Corp, Chicago, IL, USA). P values less than 0.05 were considered as significant. Quantitative variables were described using mean and standard deviation (SD), whereas categorical variables were expressed as ratios or percentages. Independent and paired t tests were used for comparison of FSS between two groups or within each group before and after intervention, respectively. Fisher exact test was used for comparisons when ranking the severity of fatigue (mild, moderate, and severe).

## Results

Eight patients (5 from ondansetron group and 3 from amantadine group) were excluded from the study due to non-compliance with drug therapy. The mean age (± SD) of study participants was 54.00 (± 7.88). Female/male ratio was 45/8. Thirty-nine (73.6%) of patients had fatigue as the only symptom. The frequencies of other symptoms in study participants which needed medical interventions are shown in Table 1.

**Table 1:** The frequencies of other symptoms in study participants which needed medical interventions

Symptom	Number (%)
Constipation	8 (15.1)
Spasm	5 (9.4)
Constipation + spasm	1 (1.9)
Total	14 (26.4)

Statistical analyses using student t test showed no significant difference in FSS between study groups either before treatment or after treatment (Table 2).

**Table 2:** Fatigue severity scores (mean ± SD) in amantadine and ondansetron groups before and after treatment

Time	Study groups		P value
	Amantadine	Ondansetron	
Before treatment	43.07 (± 10.36)	43.22 (± 9.67)	0.93
After treatment	37.36 (± 7.87)	40.00 (± 8.94)	0.13

However, FSS was significantly decreased in both groups after treatment (P < 0.001).

Severity of fatigue was also self-assessed as mild, moderate, or severe. Fisher exact test showed significant decrease in fatigue severity in both amantadine-treated group (P = 0.002) and ondansetron-treated group (P < 0.001) (Tables 3, 4).

**Table 3:** Fatigue severity base on patients’ self report after and before treatment with amantadine

Fatigue severity before treatment	Fatigue severity after treatment			Total
	Mild	Moderate	Severe	
Mild	3	0	0	3 (5.7%)
Moderate	5*	34	0	39 (73.6%)
Severe	0	11*	0	11 (20.8%)
Total	8 (15.1%)	45 (84.9%)	0	53 (100%)

Asterisks show significant decrease in fatigue severity after treatment

**Table 4:** Fatigue severity base on patients’ self report after and before treatment with ondansetron

Fatigue severity before treatment	Fatigue severity after treatment			Total
	Mild	Moderate	Severe	
Mild	0	0	0	0 (0%)
Moderate	3*	41	0	44 (83%)
Severe	0	4*	5	9 (17%)
Total	3 (5.7%)	45 (84.9%)	5 (9.4%)	53 (100%)

Asterisks show significant decrease in fatigue severity after treatment

While fatigue severity was not different between study groups before treatment (P = 0.21), there was a significant difference between two groups after treatments (P = 0.026) in a way that amantadine decreased severity of fatigue more than ondansetron.

We also assessed the effects of treatments in reduction of FSS to less than 28. Based on the statistical analyses, no significant difference was detected between two groups after treatments in this regard (P = 0.22) (Table 5).

**Table 5:** The effects of treatments on fatigue cure as described by FSS < 28

Fatigue cure	Study groups	
	Amantadine	Ondansetron
Yes	8 (15.1%)	4 (7.5%)
No	45 (84.9%)	49 (92.5%)
Total	53 (100%)	53 (100%)

No side effect or complication was reported following treatment with either drug.

## Discussion

Fatigue is the most frequently described symptom among MS patients and one of the most incapacitating symptoms in these patients [1]. The majority of MS patients complain from fatigue in a time point during MS course [15]. Fatigue is an independent factor associated with compromised quality of life in MS patients, after adjustment of the effects of physical disability. Consequently, anti-fatigue therapies are expected to increase quality of life in these patients [4]. Different mechanisms are involved in the pathogenesis of MS-related fatigue among them are secretion of proinflammatory cytokines, endocrine disturbances, axonal damage, and changed patterns of cerebral activation [1]. Among several pharmacological strategies to ameliorate fatigue are amantadine and ondansetron. Being primarily administered for treatment of Influenza infection and Parkinson’s disease, amantadine is the most broadly assessed drug for MS-related fatigue [1]. Numerous placebo-controlled trials reported beneficial effects of amantadine in reduction of fatigue based on subjective assessments [16]. However, based on the small sample sizes of the mentioned studies and possible biases in their designs, there is no consensus on formal prescribing of this drug in MS-associated fatigue [17]. Although data regarding the role of ondansetron in reduction of fatigue is less than amantadine, ondansetron has been successfully prescribed in patients with primary biliary cirrhosis [12] and chronic hepatitis C [13]. In the current crossover clinical trial, we compared the effects of these two drugs in reduction of fatigue in a population of Iranian MS patients. Notably, both drugs could reduce fatigue severity as reported by FSS as well as patients’ self-report. A former meta-analysis has assessed data of a parallel arms study and 4 crossover clinical trials about the effects of amantadine in MS-associated fatigue. All of them had demonstrated trivial and inconsistent amelioration of fatigue. However, the clinical significance of these reports and the influence on patient’s quality of life had not been assessed [18]. The results of current clinical trial verify the effects amantadine in improvement of this symptom and add to the sample size of the previous meta-analyses. However, the reduction in fatigue score in the current trial was not trivial. Certainly, future meta-analyses are required to provide recommendations for drug prescription.

Administration of 4 mg ondansetron twice a day in patients with chronic hepatitis C has remarkably decreased the fatigue score with more than 30% while placebo did not [13]. Such data is consistent with the results of the current study.

Although there was no significant difference in the effects of amantadine and ondansetron based on FSS, amantadine was more effective in reduction of fatigue severity according to the patients’ self-report. A previous meta-analysis reported the occurrence of amantadine-related side effects to be varied from 10 to 57% [18]. In the current study, none of drugs caused side effects or complications that needed medical intervention. Considering the negative impact of fatigue on quality of life of MS patients, our data indicates an improvement in this index in the assessed population of MS patients.

## Conclusion

Consequently, the current study demonstrates the comparable effects of amantadine and ondansetron on reduction of MS-associated fatigue as described by FSS. However, further studies in larger sample sizes are needed to verify our results. Moreover, although evidences suggest that fatigue is associated with serotoninergic pathways [13], further studies are needed to unravel the mechanisms by which these drugs affect patients’ fatigability.

## Abbreviations

MS: multiple sclerosis
FSS: Fatigue Severity Scale
MFIS: Modified Fatigue Impact Scale
SD: standard deviation

## Availability of data and materials

The analysed data sets generated during the study are available from the corresponding author on reasonable request.
